# Comparison of a Novel Handheld Telehealth Device with Stand-Alone Examination Tools in a Clinic Setting

**DOI:** 10.1089/tmj.2018.0214

**Published:** 2019-12-05

**Authors:** Nancy L. McDaniel, Wendy Novicoff, Brian Gunnell, David Cattell Gordon

**Affiliations:** ^1^ Department of Pediatrics, University of Virginia, Charlottesville, Virginia.; ^2^ Department of Public Health Sciences, University of Virginia, Charlottesville, Virginia.; ^3^ Department of Telemedicine, University of Virginia, Charlottesville, Virginia.

**Keywords:** pediatrics, telehealth, telemedicine, e-health

## Abstract

***Background and Objective:*** *Research demonstrates that telemedicine is effective in pediatric settings but little is published to validate the quality of the data acquired by remote peripheral examination devices to accurately inform clinical decision-making.*

***Introduction:*** *The primary aim was to compare a novel Food and Drug Administration (FDA)-cleared multifunctional remote examination device (Tyto) with other stand-alone digital examination devices. The secondary aim was to ascertain whether either device produced images or sounds better able to provide clinical information to clinicians caring for children.*

***Materials and Methods:*** *Otoscopic images and heart and lung sounds from 50 patients of ages 2–18 years were acquired using the novel device and a stand-alone digital otoscope and stethoscope. Data were stored on a secure server for review by physicians (two pulmonary faculty, two general faculty, two cardiology faculty, and two cardiology fellows). Reviewers were blinded and they reviewed images and audio files in a randomized manner. Images and sounds were scored in terms of quality using a Likert scale. Means and standard deviations (and* t*-tests to compare those means) were calculated. Individual (heart sounds, lung sounds, and otoscopic images) and aggregate scores were compared.*

***Results:*** *The novel device provided higher sound and image quality with less chance of an inability to make a diagnosis than the stand-alone devices. The novel device had a superior mean comparative diagnostic score with a high intra- and inter-reliability of cardiac, pulmonary, and otoscopic diagnosis.*

***Discussion and Conclusions:*** *The novel device outperformed the stand-alone digital stethoscope and otoscope and was better able to provide usable data to support a clinical encounter.*

## Introduction

Both the American Academy of Pediatrics and the Academy of Family Medicine have recommended guidelines for scheduled pediatric clinic visits.^1,2^ These visits are typically performed in an office or in a clinic setting, incorporating a history and physical examination, developmental assessment, aspects of preventative medicine, and immunizations. Assessment of the ill child may occur in the office, urgent care, or emergency department settings.^3^ There is increasing interest in the provision of these visits in remote care settings that may also include the home, school, day care, or other health care facilities. To ensure quality encounters, clinicians and patients must utilize reliable validated diagnostic equipment and streamlined methods of data collection and transfer. In recent years, digital remote examination tools, such as the digital stethoscope and otoscope, have been incorporated into clinical practice and particularly in telemedicine solutions.^4–6^

Telemedicine is the provision of health care utilizing telecommunication technologies augmented by the use of peripheral examination tools.^7,8^ Pediatric telehealth services have been incorporated into health care systems, hospitals, emergency rooms, out-patient clinics, schools, and day-care settings with evidence showing clinical effectiveness for the diagnosis and treatment of acute illness. These models often strive to replicate in-person services and as such, models have been published in the peer-reviewed literature.^9–14^ There is evidence that remote consultation can modify health practices and treatment compliance in peripheral environments for specific diseases.^15^

The introduction of a telemedicine model in suburban childcare centers using validated diagnostic tools has resulted in significantly reduced pediatric office and emergency department visits^16^ along with the additional benefit of a reduction in parental absences from work. The utilization of digital diagnostic technologies in clinical practice has particular potential in underserved and remote areas, potentially reducing interhospital transfers and waiting times to access specialty services and improved clinical outcomes for children.

The Tyto device (TytoCare Ltd., Israel) is a novel examination system that includes a built-in examination camera, an infrared thermometer, a wireless communication unit, a lithium ion battery, and a touch screen. The system also incorporates a digital stethoscope, a digital otoscope, and a tongue depressor. The Tyto platform enables the capacity for live video or store and forward applications, and users can be directed by voice- or on-screen instructions to obtain images and sounds to comport with the standard of care by enabling a remote physical examination. Neither the camera nor thermometer was used in this study.

The purpose of the study was to assess the clinical validity and reliability of a next-generation novel Food and Drug Administration (FDA)-cleared, multifunction comprehensive telehealth examination tool and compare it with the stand-alone digital peripheral examination devices currently deployed in our telehealth program. The primary aim of this study was to determine whether the novel device showed diagnostic equivalency with the commercially available stand-alone FDA-cleared telehealth examination devices routinely used in our telehealth program. There are many other devices on the market that were not studied. A secondary aim was to compare the images and sounds obtained with each of the devices to define which was best able to provide the physician with accurate clinical information. No attempt was made to make a clinical diagnosis such as aortic stenosis or otitis media from the data. The study was done to assess validity of a novel device.

## Materials and Methods

Both the novel and stand-alone devices adhere to the International Electrotechnical Commission standard for medical products and have received 510K clearance by the U.S. FDA.

The study was approved by the local Institutional Review Board with parental/guardian consent obtained in each case for participation. Data were prospectively collected from children of ages 2–18 years presenting for their scheduled visit to a Pediatric Cardiology Clinic at the University of Virginia Children's Hospital (Charlottesville, VA) affiliated with the UVA Health System and the UVA School of Medicine. A standard physical examination was performed in the clinic by faculty physicians. Once consent was obtained, the study nurse obtained heart sounds, lung sounds, and images of both tympanic membranes with the TytoCare device and the two stand-alone digital examination devices (One Digital Stethoscope (Thinklabs Medical LLC) and the Horus HD Digital Scope System (JEDMED Instrument Co). The images and sounds were randomized by device before data acquisition and subsequent review.

In each examination, the following information was recorded from each participant:

- Four heart sounds from the novel device and four from the stand-alone stethoscope.
- Six lung sounds from the novel device (front/back of body) and six from the stand-alone stethoscope.
- Two ear images from the novel device (left/right) and two from the stand-alone otoscope.

All data were loaded onto a secure server. The sounds and images were reviewed by eight physicians (two fellows in cardiology, two pediatric pulmonologists, two general pediatricians, and two pediatric cardiologists). The images and sounds were reviewed on a secured website and the reviewers were blind to the device and the subject.

Exclusion criteria for the study were patients with skin complaints, which might limit device use, patients with cognitive impairment, and cases wherein parental or guardian consent could not be obtained. Skin conditions such as severe inflammation might limit cooperation from discomfort. No patients who consented were excluded for skin conditions or cognitive impairment. The heart examination with each of the devices comprised recordings of the four standard auscultation points (aortic, pulmonic, tricuspid, and mitral). Lung auscultation was conducted at six standardized points (two anterior and four posterior), recording an 8 s audio at each site.

All reviewers recorded their opinions of the quality of the images and sounds on a Likert scale between 1 and 5 (where 1 = very good and 5 = very poor) such that higher mean values signified worse overall diagnostic quality. Categorical ratings were made on a scale for diagnosis at each anatomic site such that 1 = no clinical finding, 2 = significant clinical finding, and 3 = presence/absence of significant clinical finding could not be determined based upon the information provided.

### Statistical Methods

Statistical analyses were performed using IBM SPSS^®^ Version 13.0 Software (SPSS, Inc., Chicago, IL). As there were 24 recordings and images per patient (10 auscultation recordings plus 2 images with both the novel and the stand-alone devices), a minimum of 30 patients would be needed for the provision of 360 data points assessable by each of the measurement systems (720 total). This calculation exceeded the sample size needed to demonstrate a probability difference of 0.3 between the groups.^17^ Quality assessments of the devices were recorded as means + standard deviation with comparisons of continuous variables using the two-sampled *t*-test or the Wilcoxon rank sum test where appropriate. Chi-squared, Fisher's exact testing, and proportional Z tests were used where appropriate for comparisons of quality on the categorical ratings and for diagnostic group assignment. The internal consistency of the data was measured with Cronbach's alpha and the reproducibility with the intraclass correlation coefficient.^18^ It was assumed that those who were rating the devices and measuring each data point were representative of the rating population as a whole, permitting estimates of both inter- and intrareliability, where a preset threshold >0.80 was considered good. Confidence limits of 95% were determined with *p* values <0.05 considered significant.

## Results

Between July and October 2016, 50 children were enrolled in the study that included 23 males and 27 females (mean overall age 10.5 years [range 2–17]). Of the cohort, 12 had known structural cardiac abnormalities, with 1 child having a history of arrhythmia. The remainder of the children were referred because of a range of conditions including murmur, chest pain, palpitations, and syncope.

Images and sounds obtained with the novel device were judged as of higher quality when compared with those obtained with the stand-alone peripheral device (mean quality score overall = 2.8 + 1.05 vs. 3.39 + 0.94, respectively, where smaller means equaled better quality; *p* < 0.001). *Table 1* gives comparisons between the novel and the stand-alone device with regard to image and sound quality. The novel device was more likely to be rated higher overall, with less chance that the clinician was unable to document a clinical finding when using the device (*p* = 0.001) as compared with the stand-alone devices. Both the Cronbach alpha and intraclass correlation coefficients for both inter- and intrareliability exceeded the preset threshold of 0.80 (0.84–99, 0.90–0.99, respectively). *Table 2* gives similar comparisons for clinical findings, in which the novel device was more likely than the stand-alone devices in enabling the clinicians to detect a clinical finding (*p* < 0.0001) as well as for ear, heart, and lung findings, respectively (*p* < 0.0001, *p* < 0.0001, *p* = 0.004, respectively). Similarly, the Cronbach alpha and intraclass correlation coefficients for both inter- and intrareliability exceeded the preset threshold of 0.80 for diagnosis (0.85–0.99, 0.89–0.99, respectively).

**Table 1.** Statistical Summary: Image and Sound Quality Comparisons

ASSESSMENT	ANALYSIS	RECORDED DATAPOINTS	*p*
Overall image/sound quality	χ^2^ (Chi-squared)	Tyto 1,381	0.0001
		UVA^a^ 1,511	
Proportions without a diagnosis	Proportional *Z*-test	Tyto 0.53	0.001
		UVA 0.48	
Image/sound quality mean score (SD)	Two sample *t*-test	Tyto 2.80 (1.05)	0.0001
		UVA 3.39 (0.94)	
Image quality—ear	χ^2^ (Chi-squared)	Tyto 696	0.0001
		UVA 797	
Sound quality—heart	χ^2^ (Chi-squared)	Tyto 350	0.0001
		UVA 381	
Sound quality—lung	χ^2^ (Chi-squared)	Tyto 335	0.0001
		UVA 353	
Image/sound quality—inter-rater reliability	Cronbach alpha/intraclass correlation coefficient	0.84–0.99	
Image/sound quality—intra-rater reliability	Cronbach alpha/intraclass correlation coefficient	0.90–0.99	

^a^ Auditory comparisons were made with the One Digital Stethoscope (Thinklabs Medical LLC) and imaging comparisons were made with the Horus HD Digital Scope System (JEDME Instrument Co). Both are listed as standard of care UVA devices used in clinical practice.

SD, standard deviation.

**Table 2.** Statistical Summary: Diagnostic Comparisons

ASSESSMENT	ANALYSIS	RECORDED DATAPOINTS	*p*
Overall diagnosis	χ^2^ (Chi-squared)	Tyto 1,378	0.0001
		UVA 1,498	
Proportions without a diagnosis	Proportional *Z*-test	Tyto 0.56	0.001
		UVA 0.48	
Diagnostic mean score	Two sample *t*-test	Tyto 1.66	0.0001
		UVA 1.95	
Diagnosis—ear	χ^2^ (Chi-squared)	Tyto 694	0.0001
		UVA 774	
Diagnosis—heart	χ^2^ (Chi-squared)	Tyto 350	0.0001
		UVA 372	
Diagnosis—lung	χ^2^ (Chi-squared)	Tyto 334	0.004
		UVA 352	
Diagnosis—inter-rater reliability	Cronbach alpha/intraclass correlation coefficient	0.85–0.99	
Diagnosis—intra-rater reliability	Cronbach alpha/intraclass correlation coefficient	0.89–0.99	

## Discussion

This prospective cohort study from a single outpatient pediatric cardiology clinic demonstrated that the quality of images and sounds obtained using the novel device was of higher quality than those obtained using stand-alone remote examination devices routinely used in the University of Virginia telemedicine program. The novel device, which incorporates a digital otoscope, stethoscope, examination camera, and thermometer, was shown to more adequately enable remote diagnosis (no camera images or temperature data were collected in this study, so no comment can be made). There was a high level of intra- and inter-reliability for the recorded measurements of the heart, lungs, and ears. In evaluating children referred to a specialized pediatric cardiology clinic, clinicians more correctly assessed abnormal clinical findings using the novel device.

There are several issues that may be seen as limitations of this study. The participants were asked to participate on the day of a scheduled visit to the cardiology clinic. Most children were well with no cardiac pathology. It would be expected that the majority would have normal heart sounds, normal lung sounds, and normal ear examinations without significant findings. The ear images were not reviewed by an otolaryngologist and for the most part did not reveal pathology other than occasional scarring of the tympanic membrane (noted by the reviewers). Many of the ear examinations were limited by earwax that was not removed. The aim of the study was to compare the novel stand-along device with standard digital device tools. The aim of the study was not to make a diagnosis or confirm findings from the clinic visit with the novel or standard device.

The value and reliability of assessment of heart sounds with a digital stethoscope have been confirmed previously in pediatric patients.^19^ Although most murmur referrals in healthy children >1 year of age do not reveal any cardiac pathology, the ability to efficiently evaluate or follow patients using validated remote examination tools may improve access to care, triage, and reduce the burden of travel for patients and their families.^20,21^

Introduction in the 1980s of electronic stethoscopes attempted to improve sound amplification and filtration^22^ with recent implementation of noise removal algorithms capable of cancelling internally and externally derived extraneous noises likely to interfere with lower amplitude murmurs.^4^ The level of agreement with the novel stethoscope and the standard electronic device was high in our study. Given that there still remains some debate around breath sound terminology on auscultation,^23^ future definitions will influence the level of interobserver agreement for any digital device used. There are some basic practical differences between the novel and other digital stethoscopes. As an example, the One Digital Stethoscope (Thinklabs Medical LLC) has no application interface and requires the attachment of separate connectors to the audio channels, with hand manipulation of the audio filter. The hardware connections of this system have the potential to degrade the audio quality, whereas the Tyto system (which has all of its hardware in-built along with embedded filtration software) appears to have a superior sound quality and is much easier to use due to the touch screen interface.

There was high rating of the images and a high reliability with the novel device tympanic membrane diagnosis when compared with those of stand-alone digital otoscope. Our findings are in keeping with similarly reported rates using other digital equipment of enhanced visualization over conventional microscopy,^24^ with similar levels of reported diagnostic inadequacy using other digital equipment.^25^ Some of the conditions limiting the use of the digital otoscope may be device related but many issues are nondevice related (such as insufficient visualization of the tympanic membrane and/or occlusion of the ear canal with cerumen). The literature is somewhat confusing since studies are heterogeneous in their reporting of either incomplete drum visualization or lack of diagnosis when there is excess cerumen. The stand-alone digital otoscope studied provides high-resolution imagery; however, the field of view of this instrument is limited as is the distance of functioning of the automatic zoom. This invariably necessitates some manual focusing of the image. By comparison, the novel device permits a full field view of the ear canal and the tympanic membrane with preliminary white balancing of the image at the commencement of the examination and automated in-built imaging focus during the procedure.

Overall, the data concerning the efficacy of the use of digital technologies in pediatric clinical management are somewhat difficult to interpret, principally because of system variations. This may in part, however, be obviated in the future after the establishment of network groups such as the Health Experts online at Portsmouth (HELP) system in 2014, the Supporting Pediatric Research on Outcomes and Utilization of Telehealth (SPROUT) established in 2015, and consensus decision reporting for the classification of recorded lung sounds.^26,27^ The validation of diagnostic digital technology in the clinic may also provide a repository of quality sounds and images in a virtual library, which may be used for training purposes.^28^

There are many issues that impact the use of telemedicine in pediatric populations. Those issues have included reimbursement, licensure, bandwidth, electronic medical record integration, credentialing, technology choices, consumer demand, and practice guidelines.^29–33^ Equally important are concerns that care outside the context of the primary or specialty medical home, particularly when patients and their families are seeking “direct to consumer” services that may fragment care and may not favorably compare with the standard-of-care in-person visit.

In support of the use of telemedicine for pediatric populations, this study demonstrates that remote examination tools can provide high-quality data that can inform telehealth examinations. Such data may permit more widespread screening for medical conditions of childhood warranting medical attention.

## Conclusion

In summary, the novel device (Tyto) met both of the articulated study aims. In the first instance, the novel device performed better than stand-alone digital examination devices utilized in our telehealth program. In the second instance, use of the novel device resulted in lower rates of diagnostic failure with high intra- and inter-reliability for examination of the heart, lungs, and ears. In our study, the device was managed by a registered nurse with basic training in its use; there is great potential for use of the novel device by parents at home or personnel in a school or day-care facility to collect the relevant data for transmission to a remotely located clinician to inform clinical decision-making and in particular wherever possible within the context of the medical home.^34^ This approach may augment care of children with special needs or medical fragility.^35^
